# Evidence for the healthy immigrant effect in older Chinese immigrants: a cross-sectional study

**DOI:** 10.1186/1471-2458-14-603

**Published:** 2014-06-14

**Authors:** Laura Corlin, Mark Woodin, Mohan Thanikachalam, Lydia Lowe, Doug Brugge

**Affiliations:** 1 Department of Civil and Environmental Engineering, Tufts University School of Engineering, Medford, MA, USA; 2 Community Health Program, Tufts University School of Arts and Sciences, Medford, MA, USA; 3 Department of Public Health and Community Medicine, Tufts University School of Medicine, 136 Harrison Avenue, Boston, MA 02111, USA; 4 Executive Director of the Chinese Progressive Association, Boston, MA, USA

**Keywords:** Healthy immigrant effect, Chinese immigrants, Convergence of health status

## Abstract

**Background:**

Previous work has found that first-generation immigrants to developed nations tend to have better health than individuals born in the host country. We examined the evidence for the healthy immigrant effect and convergence of health status between Chinese immigrants (n = 147) and U.S. born whites (n = 167) participating in the cross-sectional Community Assessment of Freeway Exposure and Health study and residing in the same neighborhoods.

**Methods:**

We used bivariate and multivariate models to compare disease prevalence and clinical biomarkers.

**Results:**

Despite an older average age and lower socioeconomic status, Chinese immigrants were less likely to have asthma (OR = 0.20, 95% CI = 0.09–0.48) or cardiovascular disease (OR = 0.44, 95% CI = 0.20–0.94), had lower body mass index (BMI), lower inflammation biomarker levels, lower average sex-adjusted low-density lipoprotein (LDL) cholesterol, and higher average sex-adjusted high-density lipoprotein (HDL) cholesterol. However, there was no significant difference in the prevalence of diabetes or hypertension. Duration of time in the U.S. was related to cardiovascular disease and asthma but was not associated with diabetes, hypertension, BMI, HDL cholesterol, LDL cholesterol, socioeconomic status, or health behaviors.

**Conclusions:**

The lower CVD and asthma prevalence among the Chinese immigrants may be partially attributed to healthier diets, more physical activity, lower BMI, and less exposure to cigarette smoke. First generation immigrant status may be protective even after about two decades.

## Background

The health of Chinese immigrants is of public health importance since individuals from China represent the second largest immigrant group with over two million people residing in the U.S. [1]. Much of the immigrant health research focuses on the healthy immigrant effect and convergence of health status. According to these theories, immigrants tend to have better health than native-born residents initially, but with increased time in the host country, immigrants’ health approaches that of native-born residents [2-6]. These trends are observed despite the fact that first generation immigrants often have a lower average socioeconomic status (SES) than native-born residents, potentially due to some combination of a self-selection bias, underdiagnoses of health conditions prior to immigration, healthier ingrained behaviors, a likelihood of returning to their host country upon becoming sick, or lower susceptibility [3,6-9].

While the healthy immigrant effect seems to be common, specific health trends vary among immigrants from different countries [10]. Most of the national surveys, however, are aggregated by ethnicity so information is limited about specific groups [11]. Among work that has considered Chinese immigrants specifically, there is some evidence that Chinese immigrants have a lower asthma prevalence [12,13] and overall better general health [14,15]. Additionally, some cardiovascular risk factors may be associated with duration in the U.S. [16]. However, conflicting evidence exists about whether Chinese immigrants have lower diabetes prevalence than native-born residents [17,18].

Given the relative paucity of research on the health profile of Chinese immigrants in the U.S. and inconsistencies among studies that have considered this group, we sought to investigate differences in health status between Chinese immigrants and U.S. born whites participating in the Community Assessment of Freeway Exposure and Health (CAFEH) study. We also wanted to assess whether there was evidence of converging health status with increasing length of residence time in the U.S.

## Methods

**Figure 1:**
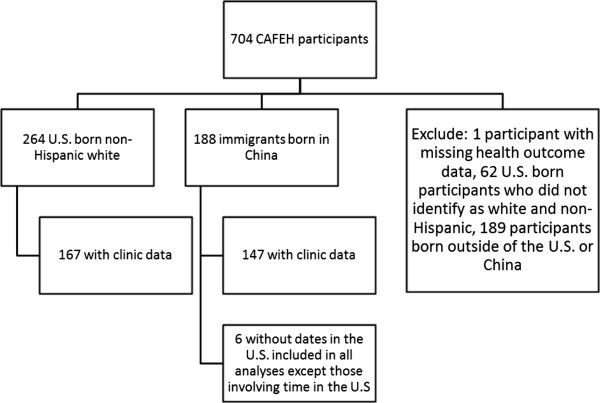
Participant selection.

CAFEH is a cross-sectional community based participatory research study of the relationship between highway related air pollution and cardiovascular health. More detailed explanations of the methods for the CAFEH study have been given elsewhere [19,20]. Participants were at least 40 years of age and were recruited from three proximity strata within three study areas in and near Boston (≤100 m of either Interstate 90 or Interstate 93, 100-450 m from either highway, or >1000 m from either highway). A stratified random sample of addresses was obtained. Among the 901 individuals eligible to participate in the random sample, the response rate was 52.4 percent for the surveys. Among these participants, 274 gave blood samples at the clinic. A convenience sample was also recruited consisting of residents in four elderly housing developments, two each in Somerville and Dorchester (n = 128) and addresses from the same buildings and floors as our random sample in Chinatown (n = 104).Overall, CAFEH had 704 participants. For this sub-analysis, only participants who self-identified as white, non-Hispanic and born in the U.S. (n = 265) or who stated that they were of Chinese ethnicity and born in China (n = 188) were included. One U.S. born white participant was excluded due to missing self-reported health outcome data. Among the 452 participants for whom clinic blood values were available, there were 167 U.S. born white and 147 Chinese participants. Thus, the sample analyzed included 314 participants, 58 percent of whom were part of the random sample (Figure 1). While the CAFEH study included participants from many races and ethnicities, we compared these groups because numbers in other sub-groups were too small and because the groups were recruited from the same study areas with the same inclusion/exclusion criteria. The largest other racial/ethnic groups in the CAFEH sample were: Hispanic (n = 51), U.S. born black (n = 40), foreign born Vietnamese (n = 33), and foreign born black (n = 24). The participants recruited from the random sample and the convenience sample were analyzed together because the recruitment group was not significantly associated with asthma or cardiovascular disease (CVD) in either bivariate or multivariate analysis. Data collection was identical for all participants. The Chinese immigrants in our sample lived primarily in Chinatown (n = 119) or Malden (n = 26) while the U.S. born whites lived primarily in Somerville (n = 78), Dorchester (n = 64), or Malden (n = 20).

Time in the U.S. was derived for Chinese immigrants based on the year of the interview and the year the participant reported coming to the U.S. Age at immigration was derived from age and time in the U.S. Six Chinese immigrants were excluded from all analyses involving time in the U.S. because the interview year was missing (n = 4), the immigration year was missing (n = 1), or the reported immigration year preceded their birth (n = 1). All participants provided written informed consent. This study was approved by the Institutional Review Board at the Tufts University School of Medicine.

The study team collected participant questionnaires and clinical biomarkers between 2009 and 2012. The questionnaire was administered in English, Cantonese, and Mandarin in participants’ homes for this sample. Questionnaires asked participants about a variety of factors including demographic information, health status, physical activity, diet, and perceived stress. The questionnaire for perceived stress has been validated in other studies and in our study population [21,22]. Participants who self-reported health conditions were asked to give the number of years since diagnosis (<1, 1–4, 5–9, 10–20, or >20). Medications were recorded by field staff from medication containers in the home. Height, weight, blood pressure, and blood samples were taken at up to two clinic visits but only data from the first study visit was analyzed here. Only left arm blood pressure values were used. Measurement methods for the clinic values have been described elsewhere [19,20].

### Statistical analysis

We used SPSS (Version 21) for data analysis. Time in the U.S. was analyzed for immigrants who had been in the U.S. five, ten, and fifteen years. All variables except for age, perceived stress, physical activity, and the clinic values were dichotomized for analysis of bivariate associations with odds ratios. Continuous variables were assessed for their bivariate association with nativity/ethnicity and time in the U.S. with independent samples t-tests. Clinical biomarkers were assessed for their sex and age-adjusted association with nativity/ethnicity. Number of minutes of exercise per week, C-reactive protein (CRP), interleukin-6 (IL-6), and tumor necrosis factor α-receptor II (TNFα-RII) were log transformed due to the skewed distributions.

Certain variables were derived from multiple questions. Participants were classified as exposed to secondhand smoke (SHS) if they were exposed in either the home or car. Participants who ate at least seven weekly servings of food were considered frequent consumers. Participants were classified as hypertensive if they had a measured systolic blood pressure above 140, diastolic blood pressure above 90, or if they reported taking medications that treat hypertension.

The primary health outcomes were self-reported doctor-diagnosed asthma, CVD, and diabetes. CVD was defined as at least one of self-reported doctor-diagnosed angina, previous stroke (type unspecified), or previous heart attack. We used the combined CVD category rather than the individual cardiovascular outcomes due to the small number of total events and the similarities in the underlying pathophysiology (atherosclerosis) [23,24]. Participants who self-reported doctor-diagnosed congestive heart failure were analyzed separately. Participants who either self-reported doctor-diagnosed diabetes or who were taking medications that treat diabetes were classified as diabetic.

We built linear regression models for asthma and CVD. We used correlation matrices to identify variables associated with the outcomes (*p* < 0.15) and to ensure that the variables included were not collinear (*p* < 0.05 and r > 0.50). The Nagelkerke R^2^, negative two log likelihood statistic, the Hosmer-Lemeshow statistic, classification tables, standardized residuals, and the effect on the standard error and *p* value for each variable already in the model were assessed after the removal of each other variable. To check these models, we used a forward selection stepwise process.

## Results

### Differences in health profile by nativity and ethnicity

Of the 314 participants, 59.2 percent were female (n = 186; Table 1). Participants ranged in age from 40 to 91 (mean 62.4 years). Chinese immigrants were significantly older and were more likely to have a lower SES than U.S. born white participants. Chinese immigrants were less likely to be current smokers, former smokers, or be exposed to cigarette smoke than the U.S. born whites (OR = 0.31, 95% CI = 0.16–0.60; OR = 0.21, 95% CI = 0.12–0.37; OR = 0.34, 95% CI = 0.18-0.66). Overall, females were less likely to be either current smokers (OR = 0.37, 95% CI = 0.20–0.66) or former smokers (OR = 0.27, 95%CI = 0.16–0.46). This difference was due to the sex differences among the Chinese immigrants. Of the 91 female Chinese immigrants, only one was a current smoker and one was a former smoker. Among the U.S. born whites, there were no significant differences in smoking status by sex.

**Table 1:** Demographic characteristics and health status

	**Total (%, n)**	**U.S. Born white (%, n)**	**Chinese overall (%, n)**	**OR**	**95% CI**
Female	59.2 (186)	56.9 (95)	61.9 (91)	1.23	0.78 - 1.94
Age*	62.4 (12.8)	59.8 (11.9)	65.3 (13.3)	-3.88	-8.36 - -2.73
High School Graduate	64.4 (199)	87.4 (146)	36.1 (53)	0.08	0.05 - 0.14
College Graduate	22.9 (72)	37.1 (62)	6.8 (10)	0.12	0.06 - 0.25
Income ≤ $25 K	61.7 (179)	34.2 (53)	93.3 (126)	26.94	12.69 - 57.23
Employed at least part time	36.0 (112)	44.2 (73)	26.7 (39)	0.46	0.29 - 0.74
Current Smoker	17.9 (56)	25.3 (42)	9.5 (14)	0.31	0.16 - 0.60
Former Smoker**	38.1 (98)	56.0 (70)	21.2 (28)	0.21	0.12 - 0.37
Never Smoked	51.1 (160)	32.5 (54)	72.1 (106)	5.36	3.30 - 8.71
SHS in Home	10.2 (31)	12.7 (21)	7.2 (10)	0.53	0.24 - 1.17
SHS in Car	11.8 (37)	18.0 (30)	4.8 (7)	0.23	0.10 - 0.54
SHS Exposed	18.2 (55)	24.8 (41)	10.1 (14)	0.34	0.18 - 0.66
Frequent fruit and vegetable consumer	25.7 (76)	27.8 (42)	23.4 (34)	0.79	0.47 - 1.34
Frequent soda or sweets consumer	20.2 (62)	37.7 (61)	0.69 (1)	0.01	0.002 - 0.084
Log-light or moderate exercise (min/week)*	2.28 (0.45)	2.18 (0.45)	2.36 (0.43)	-3.37	-0.29 - -0.08
Perceived Stress Score*	3.5 (2.9)	4.3 (11.9)	2.6 (1.9)	5.65	1.13 - 2.33
CVD (angina, heart attack, stroke)	10.6 (33)	14.5 (24)	6.2 (9)	0.44	0.20 - 0.94
Angina	3.2 (10)	4.8 (8)	1.4 (2)	0.27	0.06 - 1.31
Heart Attack	7.0 (22)	9.0 (15)	4.8 (7)	0.51	0.20 - 1.28
Stroke	3.8 (12)	6.0 (10)	1.4 (2)	0.22	0.05 - 1.01
Congestive Heart Failure	5.1 (16)	9.0 (15)	0.7 (1)	0.07	0.01 - 0.53
Hypertensive	50.4 (135)	46.4 (65)	54.7 (70)	1.39	0.86 - 2.25
Diabetic	14.1 (44)	13.3 (22)	15.0 (22)	1.14	0.61 - 2.17
Asthmatic	12.7 (40)	19.8 (33)	4.8 (7)	0.20	0.09 - 0.48

* Mean, sd, t, 95% confidence interval for the difference.

**Does not include current smokers.

The Chinese also had more favorable diet and exercise behaviors. Only one Chinese immigrant reported consuming soda and none reported consuming sweets at least seven times per week. In contrast, 37.7% of U.S. born whites reported frequently consuming either soda or sweets. Furthermore, the Chinese reported a lower mean perceived stress score (t = 5.65, *p* < 0.001).

Compared to the U.S. born whites, Chinese immigrants also reported better health status on several indicators. They were less likely to have asthma (OR = 0.20, 95% CI = 0.09–0.48) or CVD (OR = 0.44, 95% CI = 0.20–0.94). There were no significant differences for hypertension or diabetes prevalence by ethnicity/nativity. The Chinese immigrants had age and sex-adjusted cytokine, BMI, and cholesterol values consistent with their better self-reported health status though there were no significant differences by nativity/ethnicity for mean triglyceride levels (Table 2).

**Table 2:** Effect of nativity/ethnicity on biomarker levels controlling for sex and age*

	**Crude level (mean, sd)**	**B**	**95% CI**
BMI	26.9 (6.4)	-0.588	-0.740 – -0.435
Log CRP	0.11 (0.5)	-0.033	-0.046 – -0.019
Log IL-6	0.15 (0.3)	-0.020	-0.029 – -0.012
Log TNFα-RII	3.4 (0.2)	-0.010	-0.015 – -0.006
Fibrinogen	467.9 (115.2)	-3.150	-6.066 – -0.234
HDL	51.3 (20.6)	0.782	0.248 – 1.315
LDL	89.0 (28.2)	-1.067	-1.843 – -0.291
Triglycerides	118.2 (76.4)	0.213	-1.785 – 2.211

*A negative value indicates that the level is lower among the Chinese immigrants than the U.S. born whites.

We built multivariate regression models assessing the most relevant risk factors for asthma and CVD (Table 3). Education, hypertension, and perceived stress were each significantly associated with asthma, independent of nativity/ethnicity. Age and diabetes status were significant factors independently associated with CVD. In our model, smoking status, sex, and BMI did not have a significant effect in the multivariate models for asthma or CVD.

**Table 3:** Logistic regression models for asthma and cardiovascular disease

**Outcomes**	**Risk factor**	**Exp(B)**	**95% CI**
Asthma	Country of birth	0.86	0.77 - 0.98
	College graduate	3.25	1.33 - 7.93
	Systolic BP >140, diastolic BP >90, or takes hypertension medication	3.40	1.42 - 8.16
	Perceived stress score	1.19	1.05 - 1.34
Cardiovascular Disease	Country of birth	0.83	0.75 - 0.92
	Age	1.09	1.05 - 1.14
	Diabetes	4.35	1.81 - 10.44

### Differences in health profile by time in the U.S

The average age of the Chinese immigrants when they came to the U.S. was 47.2 years (6–74 years, 1.4 percent immigrated as children). The Chinese immigrants had resided in the U.S. for an average of 18.4 years. Age at the time of the interview was positively associated with duration of time in the U.S. (r = 0.373, *p* < 0.001) and newer immigrants were on average 8.6 years younger. No significant differences were observed between immigrants who had resided in the U.S. for longer than ten years compared with newer immigrants in terms of SES, smoking status, exposure to SHS, diet, or amount of physical activity.

Health status, however, was better among newer immigrants with regards to self-reported cardiovascular disease and asthma. Although approximately 40 percent of the Chinese immigrants had resided in the U.S. for 15 years or less, none had ever had a cardiovascular event in that time frame while 9.6 percent (n = 8) of immigrants who had been in the U.S. for 16 or more years reported having a cardiovascular event. Furthermore, while duration in the U.S. was not significantly associated with asthma, at least four of the seven Chinese asthmatics were diagnosed since coming to the U.S. All were diagnosed between 58 and 72 years of age. Nevertheless, after controlling for age and sex, several other health status indicators including diabetes, hypertension, BMI, triglycerides, HDL, and LDL, did not differ based on time in the U.S.

## Discussion

After residing in the U.S. for an average of 18.4 years, first generation Chinese immigrants had lower self-reported chronic disease prevalence, lower clinical values for BMI, and systemic inflammation and cholesterol levels consistent with a lower disease risk than U.S. born whites residing in the same metropolitan neighborhoods. This was true despite the Chinese immigrants being older and having a lower SES. We found support for the healthy immigrant theory, but limited evidence that the health status of these immigrants converges with that of the host population over time.

We cannot directly test reasons for the healthy immigrant effect in this population since we do not have data on reasons for immigration, income or health before immigration, or data about anyone who immigrated back to China. However, consistent with one hypothesis for the healthy immigrant theory, the Chinese immigrants reported less exposure to smoking (especially among females), better diets, and more light or moderate intensity physical activity. It is likely that these healthy behaviors were culturally ingrained prior to immigration. Assuming that the Chinese immigrants developed these habits before immigrating, participants may have retained them due to the older average age at immigration (47.2 years) and living in the potentially protective ethnic enclave in Boston Chinatown. Enclaves could be protective because they facilitate access to services, food, and social opportunities [25].

The lower likelihood of exposure to smoking among the Chinese immigrants is expected based on previously published studies of Asian immigrants to the U.S. which found that immigrants were less likely to be either former or current smokers [26,27]. Other studies have found changes in smoking prevalence among younger Asian immigrants with increased acculturation to the host country [28,29]. In our sample, however, the smoking trends were similar to those observed in China (70.0 versus 52.9 percent of males, 2.3 versus 2.4 percent of females) [30].

The observed difference in the amount of leisure-time physical activity reported between the Chinese immigrants and U.S. born whites may also reflect differences in the types of activities participants are likely to self-report. Furthermore, the amount of time available for physical activity could differ since the Chinese were less likely to be employed at least part-time. Nevertheless, none of smoking exposure, diet, or exercise was significantly associated with the primary health outcomes after controlling for nativity/ethnicity.

Asthma was only significantly associated with educational attainment, hypertension, and perceived stress. Education and perceived stress were also associated with nativity/ethnicity. While the Chinese immigrants reported lower levels of perceived stress, there could be cultural differences in common coping mechanisms or tendencies to self-report stress. Nevertheless, the relatively low perceived stress could serve as a protective factor since stress is associated with an increased risk of developing an atopic disorder [31]. Concordant with Henkin et al., we did not find an association between BMI and asthma among the Chinese immigrants [32].

Our estimate that the Chinese immigrants were about one-fifth as likely to have asthma as the U.S. born whites was almost identical to a previous study of asthma prevalence among Asian immigrant children residing in Boston compared to Asian children born in the U.S. [12]. While reasons for this low (4.8 percent) asthma prevalence among the Chinese immigrants in our sample are still unclear, it is possible that environmental or social factors that differ between China and the U.S. affect the immigrants’ likelihood of having asthma. For comparison, asthma prevalence for adults in China is less than one percent [33].

Furthermore, all seven of the Chinese asthmatics in our sample were diagnosed as adults and at least four, but possibly all seven, were diagnosed since coming to the U.S. This would be unusual in the general U.S. population since the incidence of asthma is over three times higher in children than in adults (12.5/1000 versus 3.8/1000 over one year, respectively) [34]. Concordant with studies that observe asthma prevalence changes among immigrants from lower-income nations to higher-income nations, our finding is consistent with duration of residence in the U.S. being linked with asthma [35]. Alternatively, the immigrants may have acquired asthma substantially prior to diagnosis, perhaps supporting the idea that health conditions are underreported among new immigrants.

Similarly, we found support for the healthy immigrant effect with regards to CVD and limited support for the idea of increased likelihood of CVD with increased time in the U.S. There were no cardiovascular events in immigrants who resided in the U.S. for 10 years or less and only 6.1 percent of Chinese participants (n = 9) reported any events compared to 14.5 percent of the white respondents (n = 24). The low number of events was concordant with other studies of CVD prevalence among Chinese immigrants [15,36]. The difference based on duration in the U.S. could be due to the significantly younger age of the most recent immigrants.

**Figure 2:**
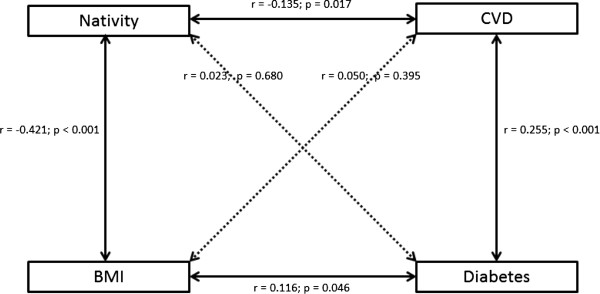
**Correlation coefficients between country of birth, BMI, CVD, and diabetes.** Lines represent statistically significant relationships. Dashed lines represent statistically insignificant relationships.

Once nativity/ethnicity was controlled for, only age and diabetes status were significantly associated with CVD. Diabetes status may be a proxy for other factors, such as BMI. While BMI was associated with diabetes, after controlling for age, education, and nativity/ethnicity, BMI was not significantly associated with CVD. Figure 2 shows these relationships. Specifically, nativity is significantly associated with both BMI and CVD status but is not significantly associated with diabetes status. BMI and CVD are not significantly associated with each other but both are significantly associated with nativity and diabetes. The relationship between BMI and CVD may be complicated since Asians have an increased likelihood of having CVD and other health effects with a BMI as low as 22 [37,38]. The difference in the effect of BMI could be due to the increased presence of body fat and central obesity at lower BMIs often seen among Asian individuals [39,40]. While our analysis used BMI as a continuous variable due to the differences in effect of moderate BMI by ethnicity, we had only 20 additional Chinese participants who would be classified as overweight using thresholds more appropriate to Chinese adults [38].

As with BMI, the association between the inflammation biomarkers and CVD is complicated by the potential for effect modification or confounding by nativity/ethnicity [41]. Previous studies indicate that log CRP is an independent risk factor for CVD [42,43]. In our sample, log CRP and log IL-6 were associated with CVD in bivariate analysis but not in the multivariate model. Nevertheless, the lower mean log CRP and log IL-6 levels among the Chinese are biological indicators of lower risk for CVD that are consistent with reported CVD outcomes.

In contrast to the trends with asthma and CVD, we did not find a significant difference in diabetes prevalence based on nativity/ethnicity. Diabetes prevalence was also not related to duration of time in the U.S. One large study of immigrants to Canada found that diabetes was associated with increased time in the host country but that study did not disaggregate data by country of origin [4]. It is possible that environmental, social, or genetic factors are responsible.

### Limitations

There were several limitations. First, the small number of cases, especially among the Chinese immigrants for some health outcomes, limited our ability to control for potentially important risk factors. Second, we only had self-reported measures for several indicators potentially limiting the sensitivity of our measures. For example, the diet questions asked about the frequency of consuming broad categories of food and did not assess portion sizes. Since we did not have blood sugar levels, we may have incorrectly classified some individuals as non-diabetic. Additionally, Asian populations, and especially older Asian populations, may be unlikely to report physical or mental health symptoms so the health status of the Chinese participants may be worse than reported. Third, we compared the Chinese immigrants only to U.S. born whites. Disparities between the ethnic groups in the effect of the various biomarkers could limit the comparability of the groups. A comparison of first and second generation Chinese immigrants would be instructive, as would a comparison of first generation Chinese immigrants to other ethnic populations in the study area. The generalizability of our results may be limited by selection bias, especially for participants in the convenience sample. Furthermore, we do not know the temporal relationship between any of the risk factors and the health outcomes and we do not know about individuals’ health status changes over time. Additionally, the mean length of time the immigrants had resided in the U.S. was only 18.4 years and we did not have data on the participants’ level of acculturation so potential health changes over several decades or changes with increasing acculturation could not be assessed. Finally, we did not have information about the immigrants’ family history of disease or information about the immigrants’ environment prior to coming to the U.S. Specifically, while we know that most of the immigrants were from South China, we do not know whether they were from urban or rural areas. Without an analysis of these and other relevant environmental or social factors affecting the participants, we cannot fully address the reasons for the differences in health status.

## Conclusions

We found that first generation immigrant status seems to be protective in terms of asthma, CVD, BMI, inflammation biomarker levels, and cholesterol levels for Chinese immigrants, even after almost two decades in the U.S. This could be because the Chinese immigrants retained healthier lifestyles. However, the health advantage does not extend to all chronic diseases. Future research should examine social and environmental risk factors that contribute to differences in health outcomes among immigrant populations.

## Abbreviations

BMI: Body mass index; CAFEH: Community assessment of freeway exposure and health; CRP: C-reactive protein; CVD: Cardiovascular disease; IL-6: Interleukin-6; LDL: Low-density lipoprotein; HDL: High-density lipoprotein; SES: Socioeconomic status; SHS: Secondhand smoke; TNF: Tumor necrosis factor α-receptor II.

## Competing interests

Laura Corlin has received travel support to work with the non-profit Possible Health (formerly known as Nyaya Health) in Nepal from Morgan Stanley. Doug Brugge has received travel support from International Physicians for the Prevention of Nuclear War to speak about the health hazards associated with uranium mining. No other authors have conflicts of interest to declare.

## Authors’ contributions

LC completed the analyses and led the writing. MW assisted with the analyses and interpretation of data. MT and LL assisted with the interpretation of data. LL also served on the steering committee that directed the CAFEH study and helped guide recruitment for the Chinatown neighborhood. DB helped conceive, directed, and supervised the CAFEH study and the analysis presented here. All authors approved the final version.

## Authors’ information

LC is a graduate student at Tufts University advised by MW and DB. MW, MT, and DB serve on the faculty at Tufts. DB directs the CAFEH study. LL is a community partner from Chinatown and member of the CAFEH steering committee.

## Pre-publication history

The pre-publication history for this paper can be accessed here:

http://www.biomedcentral.com/1471-2458/14/603/prepub
